# Antigen-Specific IgG ameliorates allergic airway inflammation via Fcγ receptor IIB on dendritic cells

**DOI:** 10.1186/1465-9921-12-42

**Published:** 2011-04-10

**Authors:** Yumiko Ishikawa, Kazuyuki Kobayashi, Masatsugu Yamamoto, Kyosuke Nakata, Tetsuya Takagawa, Yasuhiro Funada, Yoshikazu Kotani, Hajime Karasuyama, Masaru Yoshida, Yoshihiro Nishimura

**Affiliations:** 1 Division of Respiratory Medicine, Department of Internal Medicine, Kobe University Graduate School of Medicine, Kobe, Japan; 2 Division of Metabolomics Research, Division of Gastroenterology, The Integrated Center for Mass Spectrometry, Kobe University Graduate School of Medicine, Kobe, Japan; 3 Department of Immune Regulation, Tokyo Medical and Dental University Graduate School, Tokyo, Japan

## Abstract

**Background:**

There have been few reports on the role of Fc receptors (FcRs) and immunoglobulin G (IgG) in asthma. The purpose of this study is to clarify the role of inhibitory FcRs and antigen presenting cells (APCs) in pathogenesis of asthma and to evaluate antigen-transporting and presenting capacity by APCs in the tracheobronchial mucosa.

**Methods:**

In FcγRIIB deficient (KO) and C57BL/6 (WT) mice, the effects of intratracheal instillation of antigen-specific IgG were analysed using the model with sensitization and airborne challenge with ovalbumin (OVA). Thoracic lymph nodes instilled with fluorescein-conjugated OVA were analysed by fluorescence microscopy. Moreover, we analysed the CD11c^+ ^MHC class II^+ ^cells which intaken fluorescein-conjugated OVA in thoracic lymph nodes by flow cytometry. Also, lung-derived CD11c^+ ^APCs were analysed by flow cytometry. Effects of anti-OVA IgG1 on bone marrow dendritic cells (BMDCs) *in vitro *were also analysed. Moreover, in FcγRIIB KO mice intravenously transplanted dendritic cells (DCs) differentiated from BMDCs of WT mice, the effects of intratracheal instillation of anti-OVA IgG were evaluated by bronchoalveolar lavage (BAL).

**Results:**

In WT mice, total cells and eosinophils in BAL fluid reduced after instillation with anti-OVA IgG1. Anti-OVA IgG1 suppressed airway inflammation in hyperresponsiveness and histology. In addition, the number of the fluorescein-conjugated OVA in CD11c^+ ^MHC class II^+ ^cells of thoracic lymph nodes with anti-OVA IgG1 instillation decreased compared with PBS. Also, MHC class II expression on lung-derived CD11c^+ ^APCs with anti-OVA IgG1 instillation reduced. Moreover, in vitro, we showed that BMDCs with anti-OVA IgG1 significantly decreased the T cell proliferation. Finally, we demonstrated that the lacking effects of anti-OVA IgG1 on airway inflammation on FcγRIIB KO mice were restored with WT-derived BMDCs transplanted intravenously.

**Conclusion:**

Antigen-specific IgG ameliorates allergic airway inflammation via FcγRIIB on DCs.

## Background

It is estimated that as many as 300 million people of all ages suffer from bronchial asthma, and that asthmatic patients are increasing by 50% per decade worldwide [1]. The mucosa of respiratory tracts are replete with organized follicles and scattered antigen reactive or sensitized lymphoid elements, including B cells, T cells, plasma cells, dendritic cells (DCs) and a variety of other cellular elements against invading pathogens. The mucosal surfaces are also known to possess critical immunoglobulins, such as IgA, IgM and IgG.

Bronchial asthma is characterized by allergic inflammation of the bronchial mucosa, in addition to airway hyperresponsiveness (AHR), and elevated titers of circulating IgE. In asthmatic patients, antigen-specific IgE binds to FcεRI on mast cells and FcεRII on eosinophils and macrophages [2]. As a result of IgE cross-linking after antigen inhalation, an immediate allergic reaction is induced. On the other hand, the T helper 2 (Th2)-type immune response plays an important role in the late-phase reaction. When the inhaled allergen is recognized and presented by antigen presenting cells (APCs) in the airway, T cells are activated and differentiate from Th0 cells into Th2-type cells. Th2-type cells produce Th2 cytokines such as IL-4, IL-5 and IL-13 [3]. IL-4 activates the production of IgE in B cells, IL-5 increases the number of eosinophils in the airway, and IL-13 is involved in AHR and mucus secretion in the airway.

With regard to the study of immunoglobulins in asthma, there have been some reports on a novel anti-IgE therapy that exerts its action by reducing the amount of free IgE to bind to effector cells [4-6]. However, this approach cannot completely reduce circulating IgE, and cannot control the initial cascade of asthma pathogenesis. It is also known that IgG is present in the airway lumen and submucosa under normal conditions [7]. Although antigen-specific IgG is induced after antigen inhalation, its role in bronchial asthma remains unknown. OVA-specific IgG in rat-sera, such as IgG1 and IgG2a, is reported to increase on day 21 after OVA inhalation in asthmatic models induced by OVA [8]. Platts-Mills et al. demonstrated a progressive increase in specific serum IgG titers with extended exposure and a prevalence of Th2 cytokine-dependent IgG in cats and dogs [9]. Immunotherapy by allergen vaccination is reported to increase antigen-specific IgG titers in allergy patients [10], thus suggesting that antigen-specific IgG may exert a protective effect against allergies and bronchial asthma. However, the mechanism that antigen-specific IgG suppresses allergic airway inflammation is unclear.

There have been many studies on Fc receptors (FcRs), which are the receptors for the Fc portion of immunoglobulin [11-13]. FcRs are known to be associated with the immune responses in antibody-dependent cellular cytotoxicity or hypersensitivity [12,13]. Activating type FcRs consist of the FcR γ-chain, which has an immunoreceptor tyrosine-based activation motif (ITAM) in cytosol, while FcγRIIB is the only immunosuppressive FcR having an immunoreceptor tyrosine-based inhibitory motif (ITIM) [14-16]. FcγR and FcγRIIB on effector cells, such as macrophages or DCs regulate the immune response by influencing one another, and FcγRIIB on B cells was recently reported to negatively regulate the production of antibody [17]. FcγRIIB is present on various types of hematopoietic cells including macrophages, neutrophils and DCs [18]. DCs play an important role by presenting antigens to naive T cells in allergic airway inflammation [19-21]. Moreover, expression of FcγRs on DCs is reported to be important during the sensitization phase for the development of allergen-induced AHR and inflammation as described previously [22]. Therefore, anti-OVA IgG was intratracheally instilled during the sensitization phase in allergic murine model to analyse the role of FcRs and APCs in pathogenesis of asthma.

## Methods

### Mice

FcγRIIB-deficient [23] mice (FcγRIIB KO) of a C57BL/6J (wild type; WT) background were kindly provided by Prof. Toshiyuki Takai (Tohoku University). Mice transgenic for the OVA_323-339 _specific T cell receptor (OT-II mice) on a C57BL/6J background [24] were used. WT mice were purchased from CLEA Japan (Tokyo, Japan). All animal experiments were performed in accordance with the Guidelines for Animal Experimentation at Kobe University Graduate School of Medicine. Our research was approved by the Institutional Animal Care and Use Committee and was carried out according to Kobe University Animal Experimentation Regulations (P070703R).

### Agents

The following drugs and chemicals were purchased commercially: endotoxin-free OVA (Sigma-Aldrich, St. Louis, MO, USA); aluminium hydroxide (alum) (Sigma-Aldrich); acetyl-β-methylcholine chloride (Sigma-Aldrich); fluorescein-conjugated OVA (Invitrogen, Eugene, OR, USA); Albumin, from Bovine Serum (Wako Pure Chemical Industries, Osaka, Japan); purified rat anti-mouse CD16/CD32 antibody (BD Biosciences, Franklin Lakes, N J, USA); phycoerythrin (PE)-hamster anti-mouse CD11c antibody (BD Biosciences); biotin-mouse anti-mouse I-A[b] antibody (BD Biosciences); streptavidin allophycocyanin (BD Biosciences); Anti-MHC class II-FITC (Miltenyi Biotec, Gladbach, Germany); 7-Amino-Actinomycin D (7-AAD) (BD Biosciences); collagenase D (Roche Molecular Biochemicals, Mannheim, Germany); and deoxyribonuclease (DNase) I (bovine pancreas; Wako); ethylenediaminetetraacetic acid (EDTA), disodium salt (Wako); Roswell Park Memorial Institute (RPMI)-1640 (Sigma-Aldrich); 2-mercaptoethanol (Sigma-Aldrich); penicillin-streptomycin solution (Sigma-Aldrich); granulocyte macrophage colony-stimulating factor (GM-CSF) (Wako Pure Chemical Industries); CD4 microbeads (Miltenyi Biotec, Gladbach, Germany); and OVA peptide _323-339 _(Genway Biotech, Inc., San Diego, CA, USA).

### Establishment of anti-OVA IgG

Anti-OVA IgG1 and anti-OVA IgG2a in endotoxin-free condition were kindly provided by Hajime Karasuyama (Tokyo Medical and Dental University Graduate School). A panel of hybridomas secreting OVA-specific IgG monoclonal antibodies was prepared from splenocytes isolated from mice that immunized intraperitoneally 14 days before with OVA with injection alum plus B. pertussis toxin or complete Freund's adjuvant, as Ishikawa et al. described previously [25]. Concentrations of anti-OVA IgG1 and IgG2a were assayed by enzyme-linked immunosorbent assay (ELISA), as described previously [26].

### Sensitization and antigen challenge

Six- to ten-week-old female mice were sensitized by intraperitoneal injections of 10 μg of OVA with 1 mg of alum on days 0, 7 and 14. They were then exposed to 1% OVA diluted in sterile phosphate-buffered saline (PBS) for 30 min on 2 consecutive days with an ultrasonic nebulizer (NE-U12) (OMRON, Tokyo, Japan). Each subclass of anti-OVA IgG (10 μg/50 μl) was diluted in sterile PBS and instilled intratracheally on 25th days before OVA challenge. As compared to the controls, non-specific IgG was intratracheally instilled instead of anti-OVA IgG. All mice were analyzed 24 h after the final OVA challenge.

### Bronchoalveolar lavage (BAL)

BAL fluid was obtained by instilling 0.8 ml PBS and aspirating three times (recovery >85%) on 24 h after the final antigen challenge. BAL fluid was centrifuged at 3,000 × *g *for 5 min (at 4°C), and the supernatants were stored at -80°C. Total cells in BAL fluid were counted with a hemocytometer. On cytospin preparations stained with Diff-Quick (Sysmex Corporation, Kobe, Japan), differential cell counts were determined by classification of 200 cells based on standard morphology.

### ELISA

Levels of IL-4, IL-5, IL-13 and IFN-γ in BAL fluid were determined using ELISA kits (Invitrogen, R&D systems, Minneapolis, MN, USA) according to the manufacturer's instructions. The absorbance of each sample was measured at 450 nm with iMark™ microplate reader (BioRAD, Tokyo, Japan).

### Histopathology

Murine lungs were intratracheally instilled with 10% buffered formalin at a pressure of 20 cmH_2_O for 24 h and then embedded in paraffin. Serial 4-μm sections were stained with hematoxylin and eosin (H&E).

### Measurement of airway hyperresponsiveness

At 24 h after the final aerosol challenge, AHR was assessed in conscious and unrestrained mice by means of whole body plethysmography (Buxco Electronics, Sharon, CT, USA) as described previously [27]. Each mouse was placed in a plastic chamber and exposed to aerosolized PBS, followed by increasing concentrations of aerosolized acetyl-β-methylcholine chloride solutions (methacholine) (1.56, 3.13, 6.25, 12.5, 25, 50 μg/ml) for 3 min each. Bronchoconstriction was recorded for an additional 5 min for each dose of methacholine. The highest enhanced pause (Penh) value obtained during each methacholine challenge was expressed as a ratio against the basal Penh value in response to PBS challenge.

### Analysis of OVA transport to thoracic lymph nodes

A total of 50 μl of 10 mg/ml fluorescein-conjugated OVA was intratracheally instilled in sensitized WT mice one day after intratracheal instillation of 10 μg/50 μl anti-OVA IgG1 or PBS. Thoracic lymph nodes were analyzed 24 h after OVA challenge for 30 min by fluorescence microscopy in order to verify whether anti-OVA IgG1 inhibits antigen transport to thoracic lymph nodes in allergic airway inflammation. Moreover, we analysed the CD11c^+ ^cells which intaken fluorescein-conjugated OVA in thoracic lymph nodes by flow cytometry according to previous studies [28]. Lymph nodes of OVA-sensitized and challenged mice were excised after anti-OVA IgG1 or PBS instillation. Minced lymph nodes were digested into single-cell suspensions with RPMI 1640 medium supplemented with 5% fetal bovine serum, 1 mg/ml collagenase D, 0.02 mg/ml DNase I and 5 mM EDTA for 30 min at 37°C. This was filtered through a 70-μm cell strainer. After blocking with 3% BSA in PBS, single-cell suspensions were pre-incubated with Fc-receptor blocking, purified rat anti-mouse CD16/CD32 antibodies in order to reduce nonspecific binding. PE-hamster anti-mouse CD11c antibodies, biotin-mouse anti-mouse I-A[b] antibodies and streptavidin-allophycocyanin (SA-APC) were used to identify CD11c^+ ^APCs populations in lymph nodes.

### Flow cytometry of lung-derived CD11c^+ ^cells

Lungs of OVA-sensitized and challenged mice were excised after anti-OVA IgG1 or PBS instillation. Minced lung tissues were digested and incubated with RPMI 1640 medium supplemented with collagenase D, DNase I, and EDTA. The digested cells were filtered through cell strainer, and erythrocytes were lysed with a lysing kit (Funakoshi, Tokyo, Japan). After blocking with 3% BSA in PBS, single-cell suspensions were also pre-incubated with purified rat anti-mouse CD16/CD32 antibodies. PE-hamster anti-mouse CD11c antibodies and anti-MHC class II-FITC were used to identify murine lung CD11c^+ ^APC populations.

### Preparation of BMDCs

BMDCs were differentiated from bone marrow cells in WT mice as described previously [29,30]. Cell culture medium was RPMI-1640 supplemented with penicillin-streptomycin, 2-mercaptoethanol (50 μM) and 10% FCS. BMDCs from WT mice were cultured with 20 ng/ml murine GM-CSF at day 0. On days 3 and 6, 20 ng/ml GM-CSF was added to the culture fluid. On day 6, BMDCs were employed to examine whether anti-OVA IgG1 ameliorated the activation of BMDCs.

### Effects of anti-OVA IgG1 on BMDCs *in vitro*

In order to investigate the effects of anti-OVA IgG1 on the activation of BMDCs, we cultured BMDCs in the presence of GM-CSF for 5 days. On day 6, OVA peptide (20 μM) with or without anti-OVA IgG1 (0.1 mg/ml) was added to BMDCs (2 × 10^4^) per well. On day 7, CD4^+ ^T-cells were isolated from the spleens of OT-II mice using CD4 microbeads for auto-MACS (Miltenyi Biotec, Gladbach, Germany) and CD4^+ ^T-cells (2 × 10^5^) were added to each well after washing and were incubated for 48 h. The number of viable cells was examined by colorimetric assay using the WST-8 [2-(2-methoxy-4-nitrophenyl)-3-(4-nitrophenyl)-5-(2,4-disulfophenyl)-2H-tetrazolium, monosodium salt] cell-counting kit (Dojindo, Kumamoto, Japan) according to the manufacturer's protocols. At 2 h after incubation with WST-8 solution, the absorbance of each well was measured at 450 nm with a reference wavelength of 655 nm [31].

### Transplantation of BMDCs into FcγR IIB KO mice

FcγRIIB KO mice were sensitized and challenged as described above. To verify the function of DCs in allergic airway inflammation, BMDCs (10^6 ^cells per mouse) cultured from WT mice were transplanted intravenously into FcγRIIB KO mice on day 24 before instillation of anti-OVA IgG1.

### Statistical analysis

All results are expressed as means ± standard error of the mean (SEM). A t-test was conducted in order to determine differences between two groups. As measured values were not distributed normally and the sample size was small, nonparametric analysis using a Mann-Whitney U test confirmed that differences remained significant, even if the underlying distribution was uncertain. The p values for significance were set at 0.05 for all tests.

## Results

### Intratracheal instillation of anti-OVA IgG1 ameliorated Th2 response allergic airway inflammation to a greater degree than anti-OVA IgG 2a

**Figure 1:**
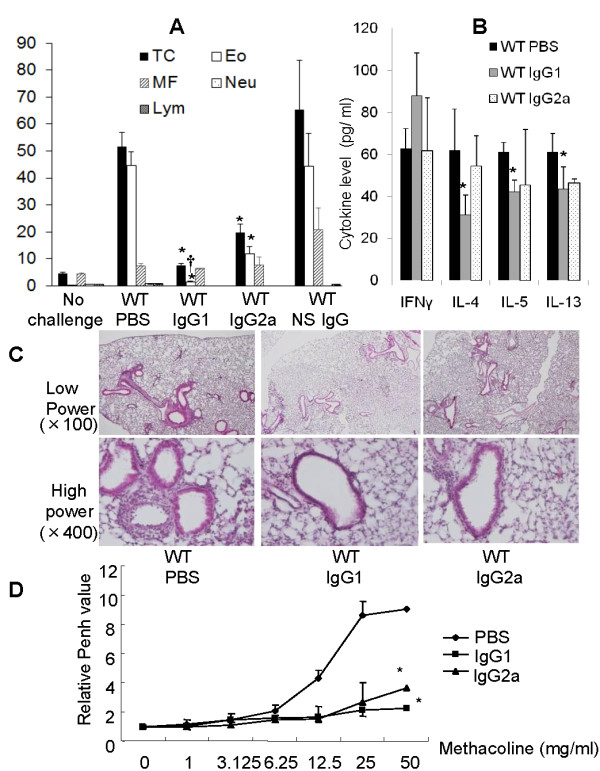
**Anti-OVA IgG inhibited allergic airway inflammation of WT mice by BAL fluid, histopathology and AHR**. A, Total cell counts and cellular composition of BAL fluid obtained from OVA-sensitized WT mice instilled with PBS, anti-OVAIgG1 and IgG2a, and non-specific IgG. *: p < 0.01 vs. WT/PBS; †: p < 0.05 vs. WT/IgG2a; TC: total cells; Eo: eosinophils; MF: macrophages; Neu: neutrophils; Lym: lymphocytes. B, Th1 and Th2 cytokine levels in BAL fluid after instillation of PBS (black bars), anti-OVA IgG1 (gray bars) or anti-OVA IgG2a (dot bars) are shown. *: p < 0.05 vs. WT/PBS. C, Histological examination of lung tissues instilled with PBS and anti-OVA IgG subclasses. Micrographs (low and high magnification, ×100 and ×400, respectively) depict lung sections stained with H&E. D, AHR after instillation with PBS and anti-OVA IgG subclasses. Increased Penh in response to inhaled methacholine was measured. Results are expressed as values relative to baseline. Data show means ± SEM pooled from three independent experiments with 4-12 mice/group.

In WT mice, anti-OVA IgG subclass instillation significantly decreased the number of total cells and eosinophils in BAL fluid as compared with PBS group, although non-specific IgG did not decrease (Figure 1A). In particular, anti-OVA IgG1 significantly attenuated the number of eosinophils in BAL fluid compared with anti-OVA IgG2a (Figure 1A). Also, IL-4, IL-5, and IL-13 in BAL fluid of anti-OVA IgG1 instillation significantly decreased compared with PBS group, on the other hand, anti-OVA IgG1 instillation increased IFN-γ cytokine levels in BAL fluid compared with PBS (Figure 1B). IL-4, IL-5, and IL-13 in BAL fluid with anti-OVA IgG2a instillation also reduced compared with PBS group, not more than anti-OVA IgG1 (Figure 1B). IFN-γ levels in BAL fluid by instillation of anti-OVA IgG2a showed no change compared with PBS group (Figure 1B). H&E-stained lung tissue sections showed increased number of eosinophils in peribronchial and perivascular areas of asthmatic WT mice (Figure 1C). Instillation of anti-OVA IgG suppressed the number of inflammatory cells in the airway compared to PBS instillation (Figure 1C). Especially, the instillation of anti-OVA IgG1 has the most suppression among every anti-OVA IgG. With regard to AHR, mice instilled with anti-OVA IgG1 also showed significant inhibition when compared to anti-OVA IgG2a (Figure 1D).

### The numbers of OVA transport to thoracic lymph nodes with anti-OVA IgG1 instillation reduced compared with PBS

**Figure 2:**
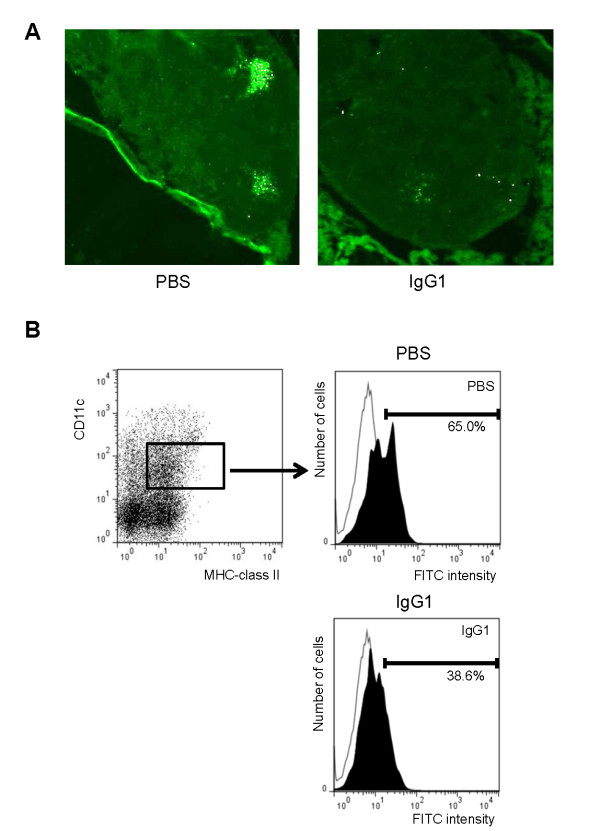
**The numbers of OVA transport to thoracic lymph nodes with anti-OVA IgG1 instillation reduced compared with PBS**. (A) Fluorescence microscopy findings of thoracic lymph nodes by instillation of OVA-fluorescein conjugate in OVA-sensitized WT mice instilled with PBS and anti-OVA IgG1. (B) The CD11c^+ ^MHC class II^+ ^cells which intaken fluorescein-conjugated OVA in thoracic lymph nodes was analysed by flow cytometry. Data show mean value pooled from three independent experiments.

In order to verify antigen transport of DCs in allergic airway inflammation, we analysed OVA transport from airway mucosa to thoracic lymph nodes by fluorescence microscopy. Fluorescein-conjugated OVA was seen in lymph nodes of allergic mice instilled with PBS (Figure 2A). Fluorescence microscopy observation confirmed a reduction in fluorescein-conjugated OVA in lymph nodes after instillation of anti-OVA IgG1 (Figure 2A). Additionally anti-OVA IgG1 also reduced the number of the fluorescein-positive CD11c^+ ^MHC class II^+ ^cells in thoracic lymph nodes compared with PBS instilled group (Figure 2B). These results show that anti-OVA IgG1 instillation in allergic inflammation reduced transport of OVA.

### MHC class II expression on lung-derived CD11c^+ ^APCs with anti-OVA IgG1 instillation reduced compared with PBS

**Figure 3:**
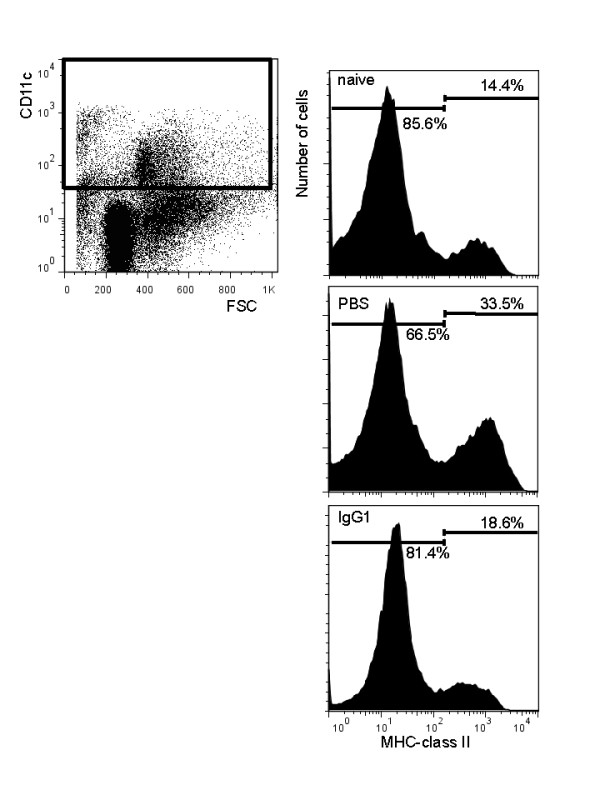
**MHC class II expression on lung-derived CD11c^+ ^APCs with anti-OVA IgG1 instillation reduced compared with PBS**. MHC class II expression on lung-derived CD11c^+ ^APCs purified from naïve mice, mice instilled with PBS or anti-OVA IgG1 were analysed. Values inside histograms represent the percentage of MHC class II expression on lung-derived CD11c^+ ^APCs. The numbers on the histogram show mean value of each percentage of gated cells from three independent experiments.

In order to clarify the role of CD11c^+ ^APCs in allergic airway inflammation, we analyzed MHC class II expression on lung-derived CD11c^+ ^APCs by flow cytometry. MHC class II expression on lung CD11c^+ ^APCs of WT mice instilled with PBS increased as compared with naïve mice (Figure 3). On the other hand, in WT mice instilled with anti-OVA IgG1, it decreased (Figure 3). These findings showed anti-OVA IgG1 instillation resulted in the decrease of MHC class II^+ ^APCs, suggesting its effects on lung APCs.

### Anti-OVA IgG1 ameliorated the activation of BMDCs *in vitro*

**Figure 4:**
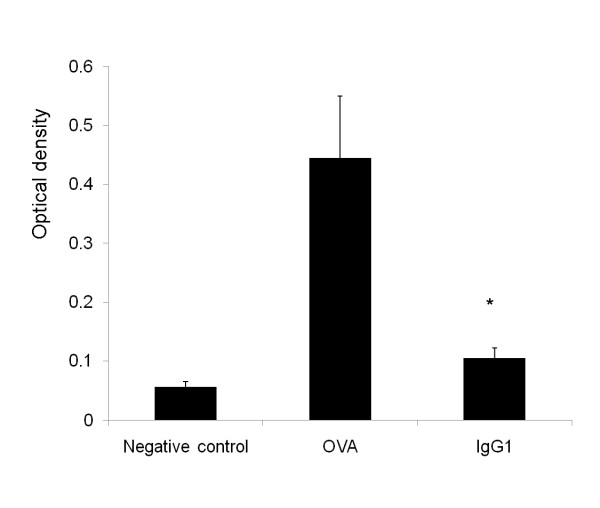
**Anti-OVA IgG1 inhibited the cell proliferation of BMDCs in vitro**. BMDCs (2 × 10^4 ^cells per well) were purified from WT mice and pulsed with OVA peptide (20 μM) or anti-OVA IgG1 (0.1 mg/ml) containing the same amount of OVA. Negative control means no addition of OVA peptide in culture medium. Cells were washed and used to stimulate CD4^+ ^T-cells (2 × 10^5 ^cells per well). The proliferation of T cells after DCs treatment with anti-OVA IgG1 was quantified in duplicate by measuring the optical density using the WST-8 cell-counting kit after 48 h. Data show means ± SEM. *p < 0.05.

The optical density of T cells, as measured with a cell counting kit, increased by addition of OVA peptide in co-culture medium with BMDCs compared to negative control (OVA peptide free). AntiOVA-IgG1 group significantly decreased the T cells proliferation (Figure 4). It means that antiOVA-IgG1 prevented BMDCs from presenting antigens, resulting in decreasing the number of T cells.

### Intratracheal instillation of anti-OVA IgG1 increases allergic airway inflammation in FcγRIIB KO mice

**Figure 5:**
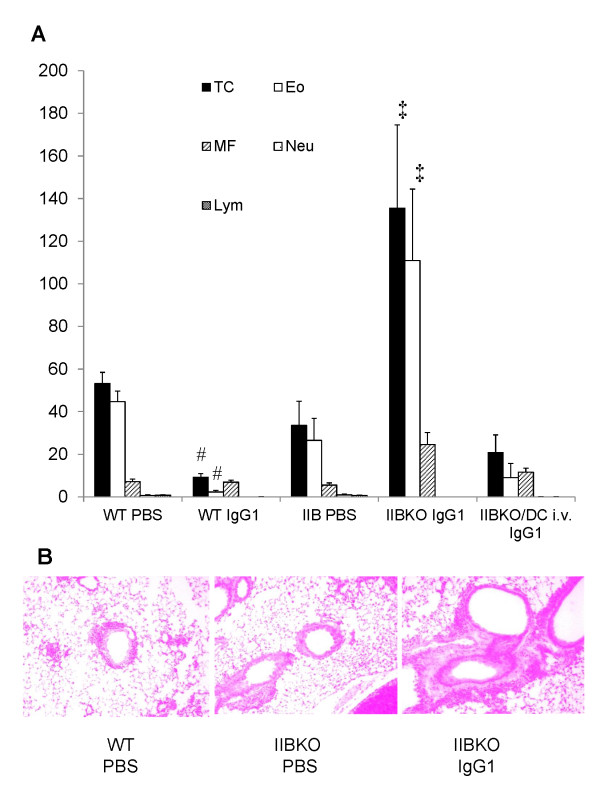
**Anti-OVA IgG1 increased allergic airway inflammation in FcγRIIB KO mice, and BMDCs transplantation reversed its effects**. A, Total cell, eosinophil and the others cell counts of BAL fluid obtained from OVA-sensitized and challenged FcγRIIB KO mice (IIBKO) instilled with PBS or anti-OVAIgG1. Moreover, FcγRIIB KO mice intravenously transplanted with WT-derived BMDCs (IIBKO/DC i.v. IgG1) were sensitized and challenged with OVA, and instilled intratracheally with anti-OVA IgG1. The number of BAL fluid was analysed. Data show mean ± SEM. #: p < 0.05 vs. WT/PBS, ‡: p < 0.05 vs. IIBKO/PBS. B, Histological examination of lung tissues of FcγRIIB KO mice instilled with PBS and anti-OVA IgG1. Micrographs (high magnification, ×400) depict lung sections stained with H&E.

Airway inflammation showed no changes in FcγRIIB KO mice instilled with PBS, similarly to WT mice, while FcγRIIB KO mice instilled with anti-OVA IgG1 significantly deteriorated airway inflammation in BAL fluid and histopathology (Figure 5A, B).

### BMDCs transplantation into FcγRIIB KO mice attenuates allergic airway inflammation by anti-OVA IgG1

In order to examine how DCs are related to allergic airway inflammation, BMDCs from WT mice were intravenously transplanted into FcγRIIB KO mice and the efficacy of anti-OVA IgG1 was analysed by BAL fluid. Total cells and eosinophils were lower in FcγRIIB KO mice transplanted intravenously and instilled with anti-OVA IgG1 compared to FcγRIIB KO mice instilled with PBS and anti-OVA IgG1, showing that WT BMDCs restored the effect of anti-OVA IgG1 on airway inflammation (Figure 5). These data indicate that anti-OVA IgG1 attenuated allergic airway inflammation via FcγRIIB in transplanted BMDCs.

## Discussion

We demonstrated that antigen-specific IgG ameliorated airway inflammation via FcγRIIB on CD11c^+ ^APCs including DCs, but non-specific IgG did not. DCs are the most potent APCs, and play a crucial role in promoting development of active immunity in allergic airway inflammation. Immature DCs sense the presence of invading antigens and process the antigen intracellularly in inflamed tissue, developing into mature DCs with upregulated expression of MHC and costimulatory molecules in inflammatory microenvironments [19-21,32]. Subsequently, mature DCs home into secondary lymphoid tissue, where they present the processed antigens to naïve T-cells to generate effector T-cells [19-21,32]. *In vitro*, there have been some reports that human IgG inhibits the differentiation and maturation of human monocyte-derived DCs [33,34]. Boruchov et al. showed that the DCs maturation marker CD83 and the costimulatory molecule CD86 on soluble human IgG-treated DCs are decreased [34]. In addition, although there are have been reports on the relationship between IgG and DCs *in vitro*, there have been no previous studies of antigen-specific IgG and DCs in murine asthmatic model. Therefore, we examined the role of DCs and the mechanisms that antigen-specific IgG suppressed allergic airway inflammation in mice.

CD11c^+ ^MHC class II^+ ^cells were previously confirmed as DCs, and were recently described as CD103^+^CD11b^- ^and CD103^-^CD11b^+ ^subpopulations [35]. We also used lung-derived CD11c^+ ^APCs to examine the mechanisms related to allergen-specific IgG and DCs. In the present study, anti-OVA IgG1 reduced the number of the fluorescein-positive CD11c^+ ^MHC class II^+ ^cells in thoracic lymph nodes compared with PBS instilled group, showing that anti-OVA IgG1 instillation in allergic inflammation reduced transport of OVA. Our findings also demonstrated that MHC class II expression on CD11c^+ ^APCs was decreased in anti-OVA IgG1 instilled mice. Moreover, WT-derived BMDCs transplantation revealed the dependence of the anti-OVA IgG1 effect on FcγRIIB on transplanted BMDCs. In addition, CD11c^+ ^DCs, not macrophages, have been reported to play crucial role in development of allergen-induced airway inflammation [21]. From above, anti-OVA IgG1 instillation suggested to modify the functions of CD11c^+ ^lung APCs, including DCs. *In vitro*, we also indicated that anti-OVA IgG1 significantly ameliorated the activation of DCs, showing a reduction of the proliferation of T cells. Our study thus demonstrated for the first time that antigen-specific IgG ameliorates the activation and maturation of CD11c^+ ^APCs including DCs in allergic mice and *in vitro*.

Mice have four different classes of FcRs; FcγRI, FcγRII, FcγRIII and FcγRIV. Functionally, FcRs are classified in two types: activating FcγRs that possess an ITAM in the cytoplasmic domain, including FcγRI, FcγRIIA, FcγRIII, FcγRIV [13,15]; and inhibitory FcγRs such as FcγRIIB, which exerts activity via ITIM. The composite expression of activating and inhibitory FcγRs thus regulates the immune response [15]. Also, in the previous studies, the presence of FcγRI-III on pulmonary macrophages and high RNA levels of FcγRIIB relative to FcγRI and FcγRIII on lung-derived CD11c^+ ^MHC class II^+ ^DCs are demonstrated [35]. In a previous study, Kitamura et al. reported that expression of FcγRs on DCs is important during the sensitization for the development of allergen-induced AHR and inflammation [22]. *In vitro *experiments, expression levels of CD86 and MHC class II were shown to be higher in BMDC in mice loaded with OVA-immune complex (IC) than in those loaded with OVA; however, no significant differences were seen in FcγR KO mice [36], suggesting more efficient maturation by OVA-IC than by OVA alone through FcγRs on DCs. Moreover, allergen-specific IgG, which is generated during sensitization, lead to IC formation upon antigen challenge and result in enhanced FcγR-mediated antigen presentation as previously described [35]. However, in our study, intratracheal instillation of anti-OVA IgG1 separately from OVA ameliorated allergic airway inflammation. This difference may be the result of differences in FcR affinity for IgG alone or IC. FcγR may more readily ligate to IC, while FcγRIIB may more readily ligate to separate IgG than IC. In the previous studies, soluble monomeric IgG decreased FcγRIIA expression and increased FcγRIIB on immature DCs in human peripheral blood mononuclear cells when soluble monomeric IgG was added to cultures of immature DCs [34]. Also, high expression levels of FcγRIIB in immature human DCs were detected, while in mature DCs FcγRIIB was markedly down-regulated as previously described [37], indicating that engagement of FcγRIIB by IC can inhibit DCs activation and antigen uptake. In mice, we suggested that FcγRIIB on immature DCs increases during sensitization by instillation of anti-OVA IgG1 and are more easily ligated with anti-OVA IgG1 alone. Moreover, Hartwig et al. demonstrated that anti-OVA IgG alone ameliorated the number of total cells and eosinophils in BAL, and peribronchial and perivascular cell counts of inflammatory cells in histology when compared with OVA-challenged mice [35]. In this point, their results correspond to our results that anti-OVA IgG alone ameliorated allergic airway inflammation and AHR, suggesting that anti-OVA IgG1 directly binds with FcγRIIB on DCs during allergic airway inflammation.

Various studies have examined the role of FcγRIIB in down-regulating specific allergic inflammatory cells *in vitro*. Dharajiya et al. also reported that FcγRIIB inhibited allergic lung inflammation in a murine model of allergic asthma, although our protocol was different from their properties of the challenged allergen or the methods of challenge. They examined the role of FcγRIIB in allergic lung inflammation using FcγRIIB KO mice sensitized to and challenged with ragweed. Ragweed challenge in sensitized mice up-regulated FcγRIIB in the lungs; however, disruption of the IFN-γ gene abrogated this up-regulation. These results indicate that ragweed challenge up-regulates FcγRIIB in the lungs via IFN-γ and Th1-dependent mechanisms. They indicated that FcγRIIB physiologically regulated allergic airway inflammation by the up-regulation of FcγRIIB on pulmonary CD14^+^/MHC class II^+ ^mononuclear cells and CD11c^+ ^cells by an IFN-γ and Th1-dependent mechanism [38]. Moreover, we have previously demonstrated that intravenous IgG ameliorated allergic airway inflammation via FcγRIIB on CD11c^+ ^DCs [39]. In another study, Sehra et al. reported that airway IgG counteracted allergen-triggered pulmonary inflammation and showed that this treatment increased Th1 reactivity and IFN-γ levels in BAL [40]. They showed that airway IgG application increased allergen capture by activating FcγR on alveolar macrophages and led to increased Th1 reactivity and IFN-γ, resulting in suppression of airway eosinophil infiltration. In this study, antigen-specific IgG was found to ameliorate antigen uptake and presentation by DCs, and suppress allergic airway inflammation via FcγRIIB, not FcγR, on DCs. In addition, anti-OVA IgG1 instillation significantly decreased IL-4, IL-5, and IL-13 cytokine levels and increased IFN-γ in BAL fluid compared with PBS. These results indicated that intratracheal instillation of anti-OVA IgG1 alone caused a shift from Th2 to Th1 in allergic airway inflammation. We indicate that anti-OVA IgG1 attenuate the activation of DCs which induces Th2 response, by binding to FcγRIIB on DCs.

We demonstrated that intratracheal instillation of antigen-specific IgG ameliorated allergic airway inflammation via FcγRIIB on CD11c^+ ^APCs including DCs. Mouse IgG1 is reported to have higher affinity for FcγRIIB than IgG2a [16]. We found that anti-OVA IgG1 most strongly ameliorated airway inflammation among the anti-OVA IgG subclasses in total cells, eosinophils, Th2 cytokine levels in BAL fluid, AHR, and histology. The present studies in FcγRIIB KO mice instilled with PBS didn't show increased inflammation compared with WT asthmatic mice. However, our previous studies demonstrated that FcγRIIB KO mice instilled with PBS showed allergic airway inflammation as WT asthmatic mice [39]. Challenged FcγRIIB KO mice instilled with PBS have increased inflammation, indicating that the balance between FcγRIIB and FcγR which existed in its cytoplasm had a tendency to FcγR of activating type FcR. Moreover, FcγRIIB KO mice instilled with anti-OVA IgG1 significantly deteriorated airway inflammation in BAL fluid and histopathology. These data suggest the balance with a further tendency to activating FcγR caused by intratracheal instillation of anti-OVA IgG1 in FcγRIIB KO.

In a murine model, intratracheally transplanted myeloid DCs from the airway are known to induce Th2 reactivity after antigen sensitization and inhalation, and leading to eosinophilic airway inflammation [41]. *In vitro*, we also indicated that anti-OVA IgG1 significantly ameliorated the activation of DCs, showing a reduction of the proliferation of T cells. Furthermore, we demonstrated that intravenous transplantation of BMDCs and intratracheal instillation of anti-OVA IgG1 ameliorated the cellular infiltration to BAL fluid compared to FcγRIIB KO mice instilled with PBS and anti-OVA IgG1, showing that i.v. transplanted BMDCs had its local effects on allergic inflammation. Our data in vivo and in vitro showed that myeloid DCs play important roles to develop Th2 response inflammation as previously reported. Our findings suggested that antigen-specific IgG ameliorated the antigen transporting and presenting capacity on CD11c^+ ^APCs including DCs, resulting in attenuating allergic airway inflammation via FcγRIIB on DCs by shifting from Th2 to Th1 immune response.

## Conclusions

We concluded that anti-OVA IgG ameliorated allergic airway inflammation via FcγRIIB on DCs in murine asthmatic model. Further studies for the function of FcγRIIB in human airway inflammation would be required. Our findings have important implications for elucidating the pathophysiology of asthmatic diseases and engineering agents to target IgG-FcR on DCs.

## Competing interests

We can state that this research is original and has not been submitted for publication elsewhere, and that no part of the research presented has been funded by tobacco industry sources.

## Authors' contributions

YI, KK, & MY designed the protocol, HK, TT, MY, & KN assisted to acquire the data, YI, MY, KK, & MY interpreted the data, YI drafted the manuscript but all of the authors contributed to the manuscript. All authors read and approved the final manuscript.
